# Defining lncRNAs Correlated with CHO Cell Growth and IgG Productivity by RNA-Seq

**DOI:** 10.1016/j.isci.2019.100785

**Published:** 2019-12-18

**Authors:** Davide Vito, Jens Christian Eriksen, Christian Skjødt, Dietmar Weilguny, Søren K. Rasmussen, C. Mark Smales

**Affiliations:** 1 Industrial Biotechnology Centre and School of Biosciences, University of Kent, Canterbury, Kent CT2 7NJ, UK; 2 Symphogen A/S, Pederstrupvej 93, DK-2750 Ballerup, Denmark; 3 AGC Biologics, Vandtårnsvej 83, DK-2860 Søborg, Denmark; 4 Alligator Bioscience AB, Medicon Village, Scheelevägen 2, 223 63 Lund, Sweden

**Keywords:** Biological Sciences, Biotechnology, Transcriptomics

## Abstract

How the long non-coding RNA (lncRNA) genome in recombinant protein producing Chinese hamster ovary (CHO) cell lines relates to phenotype is not well described. We therefore defined the CHO cell lncRNA transcriptome from cells grown in controlled miniature bioreactors under fed-batch conditions using RNA-Seq to identify lncRNAs and how the expression of these changes throughout growth and between IgG producers. We identify lncRNAs including *Adapt15*, linked to ER stress, *GAS5*, linked to mTOR signaling/growth arrest, and *PVT1,* linked to Myc expression, which are differentially regulated during fed-batch culture and whose expression correlates to productivity and growth. Changes in (non)-coding RNA expression between the seed train and the equivalent day of fed-batch culture are also reported and compared with existing datasets. Collectively, we present a comprehensive lncRNA CHO cell profiling and identify targets for engineering growth and productivity characteristics of CHO cells.

## Highlights

• The CHO cell lncRNA transcriptome is defined using RNA-Seq
• Correlations between lncRNA expression and CHO cell growth and IgG productivity found
• Expression of lncRNAs involved in ER stress correlates to productivity
• Expression of lncRNAs involved in mTOR signaling/growth arrest correlates to growth

## Graphical Abstract

**Figure:**
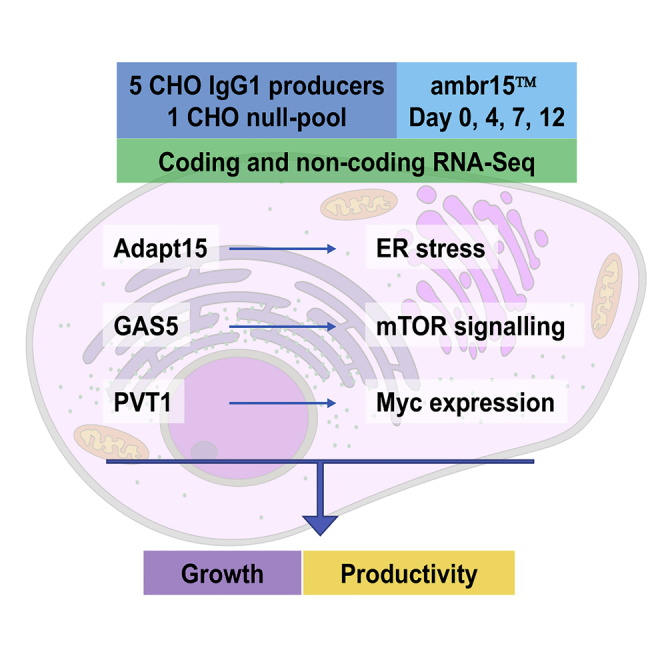

## Introduction

Many recombinant protein biopharmaceuticals are expressed in mammalian expression systems due to the ability of such systems to correctly fold, assemble, and undertake “human-like” post-translational modifications and secrete the target protein out of the cell (Walsh, 2010). Of mammalian cell expression systems one predominates, with more than 60% of mammalian made biotherapeutic proteins produced from cultured Chinese hamster ovary (CHO) cells (Kunert and Reinhart, 2016, Leu et al., 2004, Mead et al., 2015, Povey et al., 2014, Walsh, 2010). Fed-batch culture is currently the most common bioprocess used for the industrial production of proteins in CHO cells, generating increased cell concentrations (and hence biomass) and sustained culture viability compared with batch culture, ultimately resulting in higher productivity and final product yields (Durocher and Butler, 2009, Pan et al., 2017, Wong et al., 2006). The introduction of small-scale parallel bioreactors allowing automated sampling and continuous control of fundamental culture parameters, including pH, stirring, and temperature has enhanced the ability to screen a wider range of culture parameters and cell lines, leading to improved upstream development timelines and experimental throughput (Bareither and Pollard, 2011).

The ambr®15 cell culture system (Sartorius Stedim Biotech) has been shown to give similar cell growth and productivity data to those achieved in larger-scale stirred bioreactors, enabling more accurate predictions compared with shake flasks on the behavior of a cell line at larger scale (Alsayyari et al., 2018, Janakiraman et al., 2015, Nienow et al., 2013, Rouiller et al., 2016). This capacity to conduct small-scale experiments under controlled conditions, of a highly predictive nature at larger scale, allows the investigation of the behavior of different cell lines under alternative feeding regimes to determine how each respond. Indeed, recent reports state that the ambr®15 small-scale automated and controlled bioreactor system provides an excellent scale-down model to facilitate studies on multiple cell lines under controlled industrially relevant conditions to identify robust targets linked to productivity for cell engineering and material and data for future regulatory submissions (Sandner et al., 2019).

Despite advancements in the ability of CHO cells to reach higher cell concentrations and generate increasing amounts of target biotherapeutic proteins, particularly monoclonal antibodies (mAbs), there remains a desire to further understand the limitations upon CHO cell phenotypes and to engineer cells for the production of more difficult-to-express products (Godfrey et al., 2017, Jossé et al., 2018, Mead et al., 2009). One approach that has been applied toward improving our understanding of the limitations on CHO cell growth and recombinant protein production is the field of transcriptomics (Tamošaitis and Smales, 2018). Transcriptomic studies in particular could benefit from generating material from controlled miniature bioreactors that predict behavior at larger scale, as an issue of such previous studies is reproducibility and robustness across different transcriptomic datasets, given the high heterogeneity of CHO cell lines and their intrinsic genetic instability (Chen et al., 2017, Wurm, 2013, Wurm and Wurm, 2017). The availability of CHO cell and Chinese hamster genome sequences (Lewis et al., 2013, Xu et al., 2011) has greatly enabled omics-based studies (Faustrup Kildegaard et al., 2013), and since there has been an increasing number of publicly available databases for different CHO cell lines. However, the focus of these studies has been on either coding genes or microRNAs (miRNAs), with few studies investigating other classes of RNAs and their impact on CHO cell behavior (Singh et al., 2018, Tamošaitis and Smales, 2018).

Here we investigate the long non-coding transcriptome in CHO cells during fed-batch culture under controlled bioreactor conditions. Since the unraveling of multiple organisms genomes, particularly eukaryotic genomes, associated with the development of high-throughput sequencing technologies, new classes of non-coding RNA have been identified (Djebali et al., 2012). Among these, a class of transcripts known as long non-coding RNAs (lncRNAs) was identified. lncRNAs are defined as transcripts longer than 200 nucleotides that lack a significant open reading frame (ORF), are usually transcribed by RNA polymerase II, and are spliced with, or without, 3′ polyadenylation (Kashi et al., 2016, Kung et al., 2013, Wilusz, 2016). In the nucleus, *cis*-acting lncRNAs regulate the chromatin state and transcription of nearby genes, whereas *trans-*acting lncRNAs can recruit RNA-binding proteins to form chromatin-modifying complexes and modulate splicing or organize functional nuclear domains (Kopp and Mendell, 2018). When transported to the cytoplasm, lncRNAs act at a post-transcriptional level by promoting specific mRNA translation or turnover and by competitively binding microRNAs (miRNAs), attenuating the repression of target genes (Geisler and Coller, 2013). The wide range of processes involving lncRNAs suggests that some of these may be potential cell engineering targets to rewire CHO cell phenotypes for enhanced cell growth and/or recombinant protein production and quality without placing a translational burden on the cell compared with overexpression of coding genes. However, the majority of our knowledge around lncRNAs comes from studies in model organisms related to disease and development (Perry and Ulitsky, 2016, Schmitt and Chang, 2016) with lncRNAs poorly annotated in the CHO cell genome and little known about their role in defining CHO cell phenotypes.

A recent report described the lncRNA landscape in CHO cells, showing regulated expression of thousands of lncRNAs under batch and fed-batch conditions over time (Vito and Smales, 2018). Others have demonstrated the potential power of lncRNA cell engineering to manipulate the cells’ ability to produce target recombinant proteins with the engineering of a class of lncRNAs named SINEUPs enhancing the translation of target-specific mRNAs in various mammalian cell factories (Patrucco et al., 2015, Zucchelli et al., 2016). However, the limited number of studies and poor annotation of non-coding regions in the Chinese hamster genome means that transcriptomics across multiple cell lines associated with phenotypes of interest under industrially relevant and controlled conditions is required to identify lncRNAs whose manipulation may enhance mammalian cell factories’ ability to generate secreted target products (Vishwanathan et al., 2014). Here we present a comprehensive coding and non-coding, particularly lncRNA, transcriptome analysis using RNA-Seq of five IgG1 producing CHO cell lines and one stable pool harboring the plasmid cassette without genes encoding for the IgG1, cultivated under fed-batch conditions in an ambr®15 system to unveil lncRNA targets for cell engineering. The RNA-Seq datasets and analyses are made openly available to the community to promote further studies and comparisons, providing a detailed CHO cell lncRNA transcriptomic resource with a focus on particular genes and pathways.

## Results and Discussion

Following our previous landscape of lncRNA expression in CHO using microarray (Vito and Smales, 2018), we set out to provide an analysis of the lncRNA transcriptome using RNA-Seq in CHO cells producing three different model IgG1 monoclonal antibodies during fed-batch culture, defining those lncRNAs expressed in CHO cells and the flux of these during culture and between cell lines. To do this, we undertook RNA-Seq analysis on a panel of IgG-expressing CHO DHFR- cell lines (these cell lines being generated from a modified dihydrofolate reductase-deficient (DHFR-) CHO DG44 host cell line, (Urlaub et al., 1983)), sampling throughout fed-batch cultures in an ambr®15 microbioreactor system generating profiles of the flux of coding RNAs and lncRNAs.

### Analysis of Fed-Batch Culture Samples

**Figure 1:**
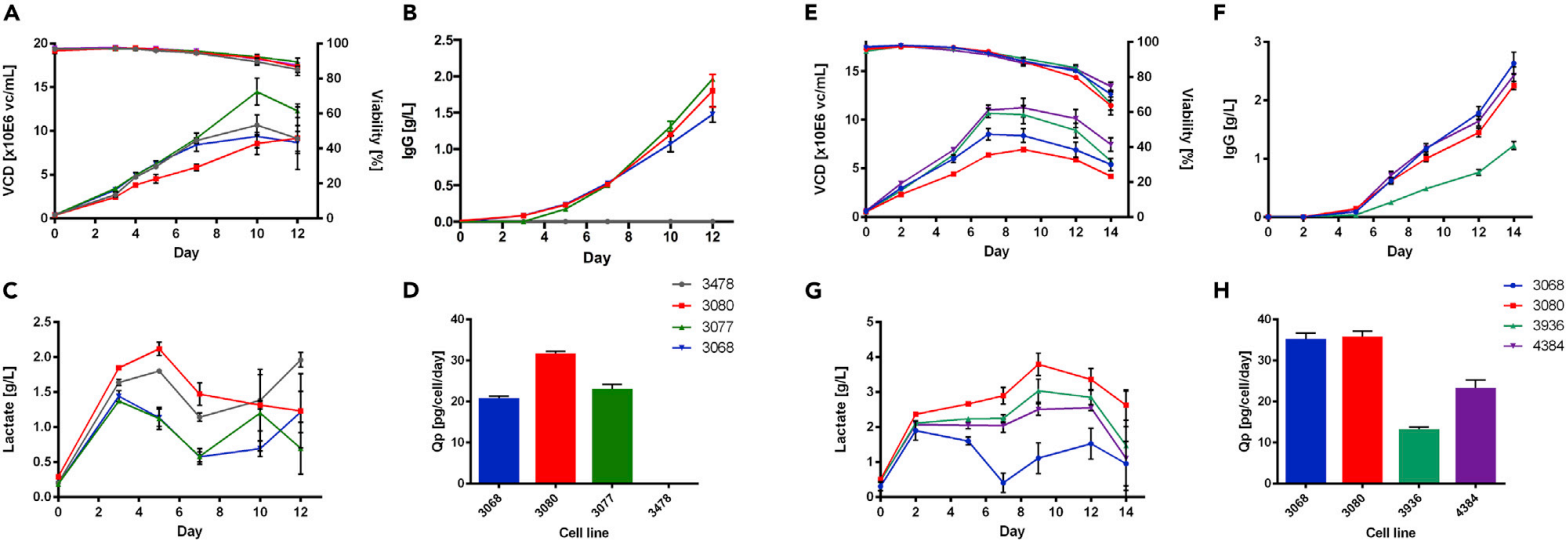
Parameters Measured and Monitored during Fed-Batch Culture of Model IgG1 Expressing CHO Cell Clones and a Null Pool The parameters measured and monitored during fed-batch culture of model IgG1-expressing CHO cell clones and a null pool for the DAVI dataset (A–D) and the JCE dataset (E–H). (A and E) Viable cell density (VCD) and viability over time. (B and F) Yield of IgG1 antibody over time. (C and G) Lactate concentration over time. (D and H) Productivity (Qp) for each cell line. Data are represented as mean ± SEM.

The DAVI dataset included the 3068, 3080 and 3077 IgG1-producing cell lines and the null pool 3478, cultured for 12 days (Table 1). These cell lines maintained culture viabilities >80% throughout the 12 days of fed-batch culture, reaching a peak in viable cell concentration (VCD) at 14.48 x 10^6^ viable cells/mL for cell line 3077, whereas 3080 had the lowest VCD throughout culture (Figure 1A). 3080 reached the highest titer at 1.80 g/L and productivity (Qp) at 31.71 pg/cell/day (3080) associated with the highest amount of lactate accumulated during the first few days of culture, with a peak concentration of 2.2 g/L (Figures 1B–1D). The glutamate, glutamine, and ammonia profiles were similar across all cell lines (Figure S1). Overall, the highest productivity was associated with the highest lactate accumulation causing the lowest VCDs for 3080, whereas the rest of the producers did not show this association compared with the null pool.

**Table 1:** Cell Line Detail/Name, Clonality, Peak Viable Cell Number (VCD), the IgG Yield at Day 12 for DAVI and Day 14 for JCE and the Cell Specific Productivity for the Model IgG1-Expressing Cells Used in the Experiments Described in the Study

Cell Line	Clonality	Peak VCD [Viable Cells/Day]	Yield [g/L]	Qp [pg/Cell/Day]
**DAVI Experiment**				

3478	Pool	10.66 x 10^6^	–	–
3068	Clone	9.38 x 10^6^	1.48	26.53
3077	Clone	14.48 x 10^6^	1.96	23.05
3080	Clone	8.56 x 10^6^	1.80	31.71

**JCE Experiment**				

3068	Clone	8.52 x 10^6^	2.64	35.20
3080	Clone	6.94 x 10^6^	2.25	35.81
4384	Clone	11.01 x 10^6^	2.43	23.39
3936	Pool	10.67 x 10^6^	1.23	13.25

The JCE dataset included the 3068, 3080, and 4384 IgG1-producing cell lines and the 3936 IgG1-producing pool, cultured for 14 days (Table 1). Once again, 3080 was the cell line showing the lowest VCDs and the highest accumulation of lactate at approximately 3.5 g/L at Day 5, whereas culture viability profiles were broadly comparable across cell lines, being within the range of 60%–80% on the last day of culture (Figures 1E and 1G). When the Qp was calculated, the 3068 and 3080 cell lines had the highest Qps at 35.20 and 35.81 pg/cell/day respectively, whereas 3936 had the lowest Qp, as would be expected from a pool of clones (Figure 1H). The glutamate, glutamine, and ammonia profiles were again broadly similar across cell lines (Figure S1). In summary, 3080 repeated the trend observed in the DAVI dataset of lowest VCD associated with highest productivity and highest accumulation of lactate, but 3068 followed closely in productivity.

### RNA Sequencing of ambr®15-Generated Samples and Subsequent Analysis of the Data: The DAVI Experiment

**Figure 2:**
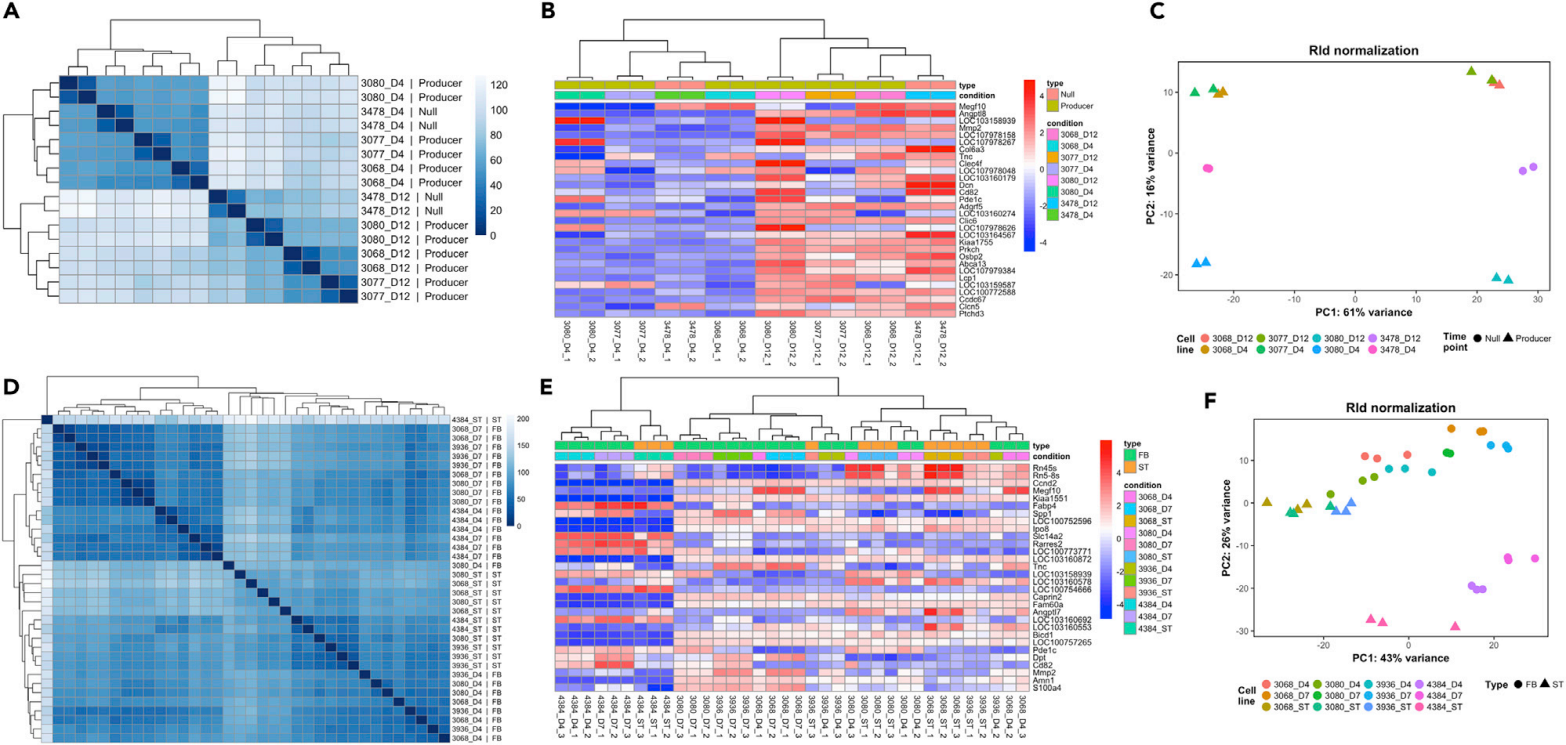
Clustering Analysis and PCA of Samples and Genes From left to right the clustering based on the distance between each sample (A and D), the clustering based on the top 30 most differentially expressed genes expression (B and E), and the PCA of normalized gene expression for the first two principal components (C and F). Panels (A–C) show the DAVI dataset. The type indicates whether the samples are from the non-producing pool (Null) or from producing clones (Producer), whereas the condition groups replicate of the same cell line and time point. Panels (D–F) show the JCE dataset. The type indicates whether the samples are from fed-batch culture (FB) or from the seed-train (ST), whereas the condition groups replicate of same cell line and time point.

As described in the methods section, samples for RNA-Seq were collected in duplicate at Day 4 and Day 12 of fed-batch culture from the ambr®15 reactors. Gene counts were calculated using featureCounts, then the differential expression analysis was undertaken with the R/Bioconductor package DESeq2 (Love et al., 2014). Clustering analysis of the RNA-Seq data revealed that the samples showed a consistent hierarchical clustering for the biological replicates and an evident separation between the two time points based on gene expression (Figures 2A and 2B). This separation was confirmed by principal component analysis (PCA); however, an additional layer of clustering emerged among the producers upon PCA with a difference in Qp, clearly showing clusters formed of 3077 and 3068 (23.05 and 26.53 pg/cell/day) that were distanced from cell line 3080 (31.71 pg/cell/day) and the null-pool 3478 (Figure 2C). Overall, this preliminary cluster analysis based on total gene expression suggests a grouping of cell lines directly related to differences in Qp more than maximum viable cell concentrations for this dataset.

**Figure 3:**
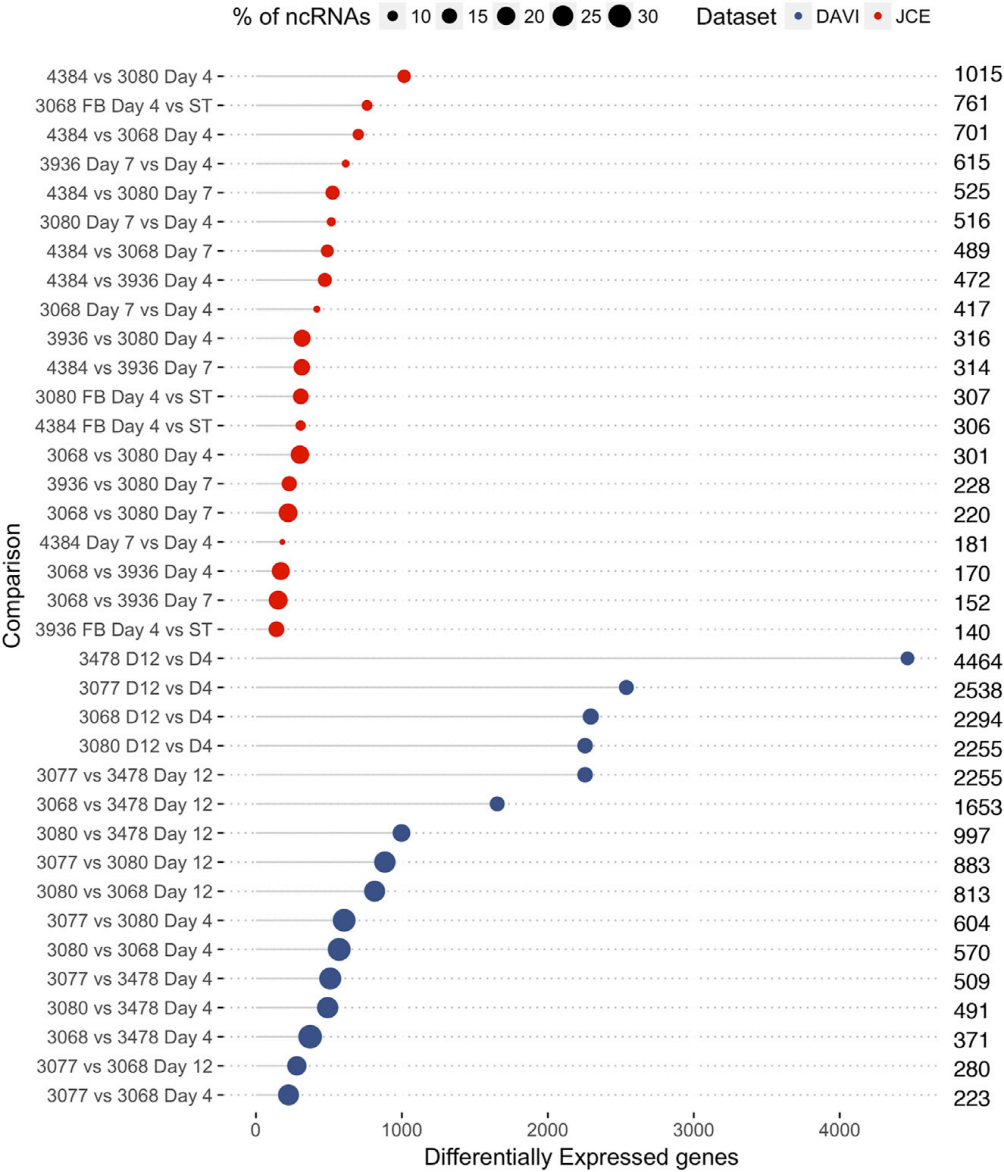
Depiction of the Number of Differentially Expressed Genes between Different Sample Comparisons The number of differentially expressed genes with an FC ≥ 1.5, *adj* p value < 0.1 (DE genes were considered significant if the adjusted p value for this fold chnage (FC) threshold calculated using the Benjamini-Hochberg method was below 0.10). In blue is shown the DAVI dataset and in red is shown the JCE dataset while the size of the dot indicates the % of lncRNAs for each comparison. For each comparison, the exact number of genes is indicated on the right.

Differential transcript expression (DE) analysis was then conducted using the DESeq2 R software package, setting a fold-change (FC) threshold of 1.50. DE genes were considered significant if the adjusted p value for this FC threshold calculated using the Benjamini-Hochberg method was below 0.10. As expected, the longest distances observed in clustering and PCA analyses corresponded to a higher number of DE genes overall (Figure 3). Consequently, samples collected at Day 12 showed the highest variability when compared between them or against Day 4 samples, with the exception of 3077 vs 3068, which indeed clustered together in PCA (Figure 2). Within the identified DE genes, lncRNAs made up 10–30% of the total number identified (Figure 3). A representative group of differentially expressed coding and non-coding RNAs were then selected (see section on identification of differentially expressed lncRNAs as potential cell engineering targets) for RT-qPCR validation, resulting in a positive correlation between the fold-changes measured by RNA-Seq and by RT-qPCR (Data S2).

### RNA Sequencing of ambr®15-Generated Samples and Subsequent Analysis of the Data: The JCE Experiment

In the JCE experiment, samples for RNA-Seq were collected in triplicate from the seed train (ST) flasks and at Day 4 and Day 7 of fed-batch culture. The samples showed a hierarchical clustering for each biological triplicate, but the separation into groups as observed in the DAVI experiment was not as evident (Figures 2E and 2F). PCA revealed a similar pattern where only a clear separated cluster composed of the 4384 cell line samples was distinguishable from the other samples (Figure 2G). The 4384 clones showed the highest VCD throughout culture (Figure 1E) and the observed distance in the PCA clustering reflected in the high number of DE genes identified when comparing this cell line against the others, especially on Day 4 (Figure 3). Although less predominant, the presence of sub-clusters grouped by time point with the same general trend can be observed among the rest of the samples (Figure 2G). We then compared the gene expression profiles of the seed-train cultures that were used to start the fed-batch process, obtained from cells during logarithmic growth phase, with the Day 4 gene expression profiles of the fed-batch culture experiments. Although cells from the seed train and fed-batch Day 4 ambr®15 bioreactor might be expected to be in a similar growth and metabolic state, we found significant differences in gene expression numbers at this early stage of culture, particularly for 3068 where the number of DE genes identified was 761 (Figure 3). Overall, the hierarchical clustering, PCA, and DE analysis suggest the 4384 cell line has a distinct transcriptional landscape, whereas 3068, 3936, and 3080 have much closer gene expression profiles. Further, the seed train samples of each cell line show, to varying degrees, different gene expression profiles than that of cells taken from the fed-batch cultures in an equivalent growth phase.

### Investigating Pathway Enrichment in DE Genes

**Figure 4:**
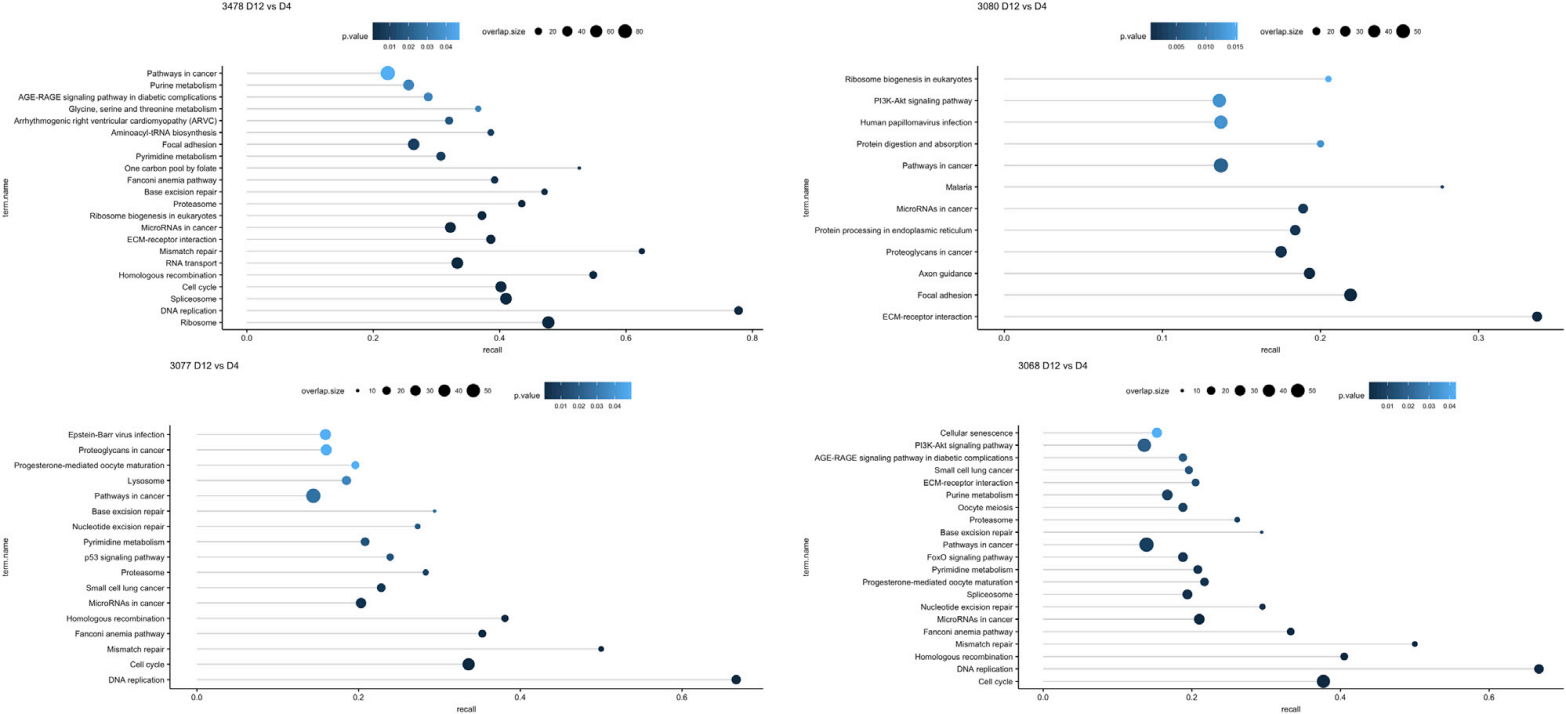
Enriched KEGG Pathways Based on Differentially Expressed Genes Enriched KEGG pathways based on differentially expressed genes for each comparison among the same cell line at Day 12 against Day 4 in the DAVI dataset. Each dot represents a pathway, with color shade representing the p value, size proportional to the overlap size (differentially expressed genes in the pathway), and x-coordinate recall (overlap size divided by the total number of genes in the pathway).

KEGG pathway functional enrichment of the RNA-Seq datasets based on statistically significant differentially expressed genes showed two distinct patterns across the datasets. Firstly, a major theme of enrichment in the DAVI dataset was in the Replication and Repair area, where DE genes were found to be enriched in DNA replication, base excision repair, nucleotide excision repair, mismatch repair, homologous recombination, and Fanconi anemia pathways among the 3077, 3068, and 3478 cell lines when comparing Day 12 versus Day 4 expression profiles within the same cell line (Figure 4). Interestingly, the only cell line in the DAVI dataset in which none of these pathways was enriched was the 3080 cell line. In the DAVI dataset this was the cell line with highest Qp associated with lowest VCD and a clear distinction from the others when the RNA-Seq data were analyzed by PCA. On the other hand, comparing the different cell lines to each other among Day 4 or Day 12 did not reveal any enriched pathways related to genome maintenance (Figure S2). Thus, within a given cell line changes in genome maintenance pathways were observed over time between days 4 and 12 of culture; however, when same time points of different cell lines were compared this was not observed.

We then applied the same enrichment analysis to the JCE dataset, and surprisingly none of the pathways involved in genome maintenance were enriched within the DE genes between Day 4 and Day 7 (Figure S3). An analysis of the seed train versus fed-batch DE genes for the 3080 and 3068 cell lines revealed enrichment of the DNA replication pathway and for the seed train versus 4384 cell line enrichment of the nucleotide excision repair, mismatch repair, and DNA replication pathways (Figure S4). The pathway enrichment analysis also consistently revealed the enrichment of the PI3K-Akt signaling pathway, focal adhesion, and ECM-receptor interaction pathways in the DE genes across both the DAVI and JCE datasets. Overall, the enrichment suggests a prominent regulation of genome maintenance mechanisms is conserved across different cell lines at the passage from seed train to fed-batch culture and toward the end of culture, whereas the most differentially regulated pathways at the same stage in culture for the same cell lines are the PI3K-Akt signaling, focal adhesion, and ECM-receptor pathways.

Focusing on individual genes involved in the replication and repair domain, we identified exonuclease 1 (*Exo1*), *Rad51*, essential meiotic structure-specific endonuclease 1 (*Eme1*) and FA complementation group B (*Fancb*) in common among the top 30 most differentially expressed genes in both the DAVI and JCE datasets (Figure S5). These genes are involved in a wide range of genome repair mechanisms from mismatch repair to homologous recombination and DNA double-strand break repair, suggesting a co-regulation of multiple facets of genome maintenance and the importance of high fidelity in these pathways to maintain cell integrity, viability, and growth as culture progresses.

### Mapping of Long Non-coding RNA Expression During Fed-batch Culture

As outlined in the introduction section, a key aspect of this study was to generate a detailed description of lncRNA expression under controlled bioreactor conditions in different IgG-producing CHO cells and to identify lncRNAs whose manipulation may enhance the CHO cell factory’s ability to generate secreted target products. In order to identify non-coding RNAs, all the significant differentially expressed non-coding genes were filtered based on NCBI annotation and are shown as a percentage of the total number of differentially expressed genes for each comparison in Figure 3 and in Table S1. The DAVI dataset showed a higher percentage of ncRNAs on average (22.2%) compared with JCE (14.0%) as a percentage of the total DE RNAs identified, most likely due to the higher coverage in the sequencing data for the DAVI dataset. The complete RNA-Seq dataset is provided reporting those lncRNAs identified as being expressed in CHO cells and hence providing a reference for the community to investigate individual lncRNAs in CHO cells (see GEO accession number GEO: GSE140671 and Data S1).

### Identification of Differentially Expressed lncRNAs as Potential Cell Engineering Targets

We then looked to filter and refine the list of DE lncRNAs by counting the occurrence of each transcript in all the DE comparisons, assessing sequence conservation across mammalian species through the discontiguous megablast algorithm and secondary structure prediction based on the RFAM database (Kalvari et al., 2018). Each lncRNA identified via this strategy was then experimentally validated by RT-qPCR to confirm the differential expression between conditions in CHO cells (Data S2). The first lncRNA identified via this approach was *Adapt15*. LncRNA *Adapt15* (also known as growth-arrested DNA-damage inducible gene 7, *Gadd7*) was discovered in hamster cells, with orthologs identified in the closely related *C. longicaudatus* and sequence conservation across rodents (Crawford et al., 1996). More recently, *Adapt15* has been linked to oxidative lipotoxicity, with knockdown alleviating ER stress and cell death (Brookheart et al., 2009). In the DAVI dataset at Day 12, *Adapt15* was consistently upregulated in the 3077, 3068, and 3080 cell lines compared with the null pool 3478 (Table 2). Interestingly, the lowest FC was measured in 3080, the clone with the highest Qp, suggesting Adapt15 expression could be a bottleneck for protein production when the ER is put under stress. In addition, Adapt15 is a known contributor to DNA damage, and 3080 was the only clone not showing DNA-damage-related pathway enrichment in the KEGG analysis previously described (Hollander et al., 1996). Clones with naturally lower levels of Adapt15 could be able to escape these detrimental effects on productivity and genome stability. Conversely, within the JCE dataset, which is focused only on producers and comparison of earlier stages of culture, there was no significant DE of *Adapt15* identified, suggesting DE expression of this transcript is observed in producer cell lines compared with a non-producing control later in fed-batch culture.

**Table 2:** Three Identified Potential lncRNAs Targets for Cell Engineering with an Established Function in the Literature and an Ortholog in Mouse

Gene ID	NCBI RefSeq	lncRNA	Expression in DAVI	Expression in JCE	RFAM	RFAM ID	Coding Probability	Mouse Homologue	BLAST E-value	Function
100689050	NR_045124.1	Adapt15	3068 D12 vs D4, −1.80 3080 D12 vs D4, 3.36 3478 D12 vs D4, −5.21 D12 3068 vs 3478, 2.86 D12 3077 vs 3478, 3.36 D12 3080 vs 3478, 1.88	–	–	–	0.023	NR_040384.1	5 × 10^−11^	Linked to oxidative lipotoxicity, resulting in ER stress and cell death
103158913	XR_478428.1	GAS5	3077 D12 vs D4, −1.98 3478 D12 vs D4, −2.47 D12 3068 vs 3478, 2.17	–	SNORD44 SNORD78	RF00287 RF00592	0.023	NR_002840.2	1 × 10^−65^	snoRNA host gene tumor suppressor
103158906	XR_478426.2	PVT1	3068 D12 vs D4, 1.91 3080 D12 vs D4, 3.59 D4 3077 vs 3478, 2.70 D12 3068 vs 3478, 2.71 D12 3077 vs 3478, 3.59 D12 3080 vs 3478, 2.13	D4 3068 vs 3936, 2.53 D4 3936 vs 3080, −2.63 D4 4384 vs 3080, −2.04 D7 3068 vs 3936, 2.53	PVT1_3	RF02166	0.029	NR_003368.2	2 × 10^−53^	Oncogene, interacts with miR200 family and Myc

From left to right, the table indicates the GeneID and NCBI RefSeq accessions, the gene name, the statistically significant fold-change in DAVI and JCE datasets, the RFAM secondary structure family and accession numbers, the coding probability measured in the Coding Potential Calculator 2 (CPC2) (Kang et al., 2017), the mouse homologue transcript with the corresponding E-value obtained using the disc megablast algorithm, and a summary of the biological function.

**Figure 5:**
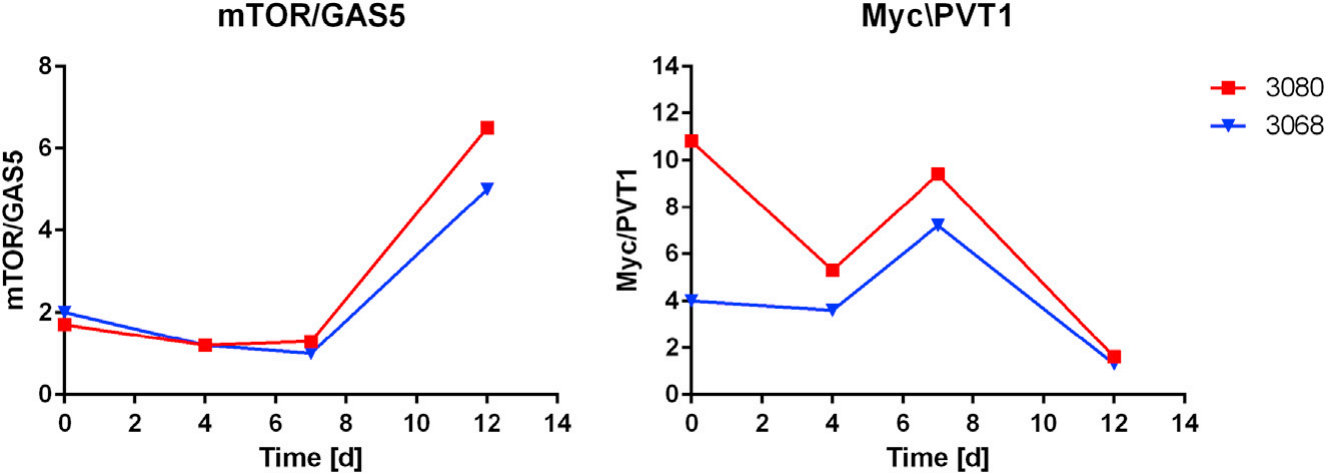
Analysis of the Expression over Time of the Two Functionally Related Genes (mRNA/lncRNA) mTOR-GAS5 and Myc-PVT1 Relative ratio or proportion of expression over time for the two functionally related genes (mRNA/lncRNA), mTOR-GAS5 (on the left) and Myc-PVT1 (on the right). These data were obtained by integrating the DAVI and JCE datasets for the 3080 (in red) and 3068 (in blue) clones. To allow the integration of the two different datasets, the proportion is reported as the gene expression ratio for the indicated gene couple at the specific time point.

A second lncRNA that showed significant DE was growth-arrest-specific transcript 5 (*GAS5*), a multiple small nucleolar RNA (snoRNA) host gene (Smith and Steitz, 1998) with a short ORF and a well-known tumor suppressor lncRNA in human cancer biology (Ma et al., 2016). As a result of cell growth arrest and mTOR pathway activity repression, translation of the *GAS5* short ORF is blocked and the transcript accumulates, escaping the nonsense-mediated decay (NMD) pathway that depends on active translation (Ma et al., 2016, Tani et al., 2013). We identified the uncharacterized gene *LOC103158913* as a ortholog of mouse *GAS5* in CHO using BLAST and RFAM predictions (Table 2). We then investigated its correlation with mTOR expression by integrating the JCE and DAVI measurements for the 3068 and 3080 producers to obtain four time points (Figure 5). We found a stable ratio between *mTOR* and *GAS5* until Day 12, when mTOR expression became predominant, especially in the highest producer 3080. In addition, *GAS5* was upregulated at Day 12 in 3068 compared with the null pool and not in the 3080, whereas *mTOR* was never differentially regulated. Taken together, literature evidence and the presented data suggest the accumulation of *GAS5* may be the result of slower translation efficiency caused by an attenuation of mTOR signaling in the later stages of culture, when recombinant protein production is at its peak, leading to lower productivity.

A third well-characterized lncRNA in other organisms that was identified in our system as differentially regulated was plasmacytoma variant translocation 1 (*PVT1*). *PVT1* is a non-coding oncogene related to poor prognosis in different cancer types (Zhu et al., 2017) with reports of competing endogenous RNA (ceRNA) activity on the miR200 microRNA family and direct interaction with the *Myc* oncogene (Colombo et al., 2015, Tseng and Bagchi, 2015, Tseng et al., 2014). PVT1 has also been found to induce PI3K/AKT cascade activation, a pathway we found consistently dysregulated in KEGG functional enrichment (Sun et al., 2019). In a previous work (Vito and Smales, 2018), we identified the uncharacterized gene *LOC103158906* as an ortholog of mouse *PVT1* in CHO using BLAST and secondary structure prediction in RFAM. *PVT1* is annotated in C_griseus_v1.0 as *LOC103162981*, giving the same BLAST alignment scores and RFAM. *Myc* proto-oncogene protein is predicted to be encoded in CHO as *LOC100758352*, containing Myc amino-terminal region (Myc_N), helix-loop-helix DNA-binding domain (HLH), and a Myc leucine zipper domain (Myc-LZ). *PVT1* expression was upregulated by more than two-fold at Day 12 in every producer compared with the null pool in the DAVI dataset and at both Day 4 and Day 7 in the JCE dataset for 3068 compared with 3936, the clone with the highest yield and the pool with the lowest yield, respectively. We then investigated the connection between *PVT1* and *Myc* by measuring the ratio of expression in the JCE and DAVI datasets for 3068 and 3080 (Figure 5). Although seed-train samples showed an initial discrepancy between the two clones, the ratio was comparable among later time points, a predominant Myc expression at Day 7, when the exponential growth phase is sustained by active proliferation, while *PVT1* expression increases substantially at Day 12 (Figure 5). When assessing the direct interaction probability of CHO *PVT1* (XR_478426.2) and *Myc* (XP_003516054.2) using lncPro and RPISeq prediction tools, the output suggested a strong likelihood of interaction, scoring 85.1 and 0.9, respectively (Lu et al., 2013, Muppirala et al., 2011). Collectively these data suggest a relationship between *PVT1*, *Myc,* and proliferation in the early stages of culture when Myc activity is preponderant and a strong overexpression of *PVT1* toward Day 12 that may promote IgG production.

### Comparison of Data Presented Here with Existing Datasets

A recent meta-analysis publication compared transcriptomics studies in CHO cells, commenting on the difficulty in comparing these datasets but also identifying the most recurrent genes identified as related to Qp and growth (Tamošaitis and Smales, 2018). Here, we find that a number of the coding genes in this study were regulated in agreement with this meta-analysis study (Data S3). The gene ranking first in the list of the meta-analysis was *Cd36*, a multifunctional glycoprotein acting as receptor for a broad range of ligands of proteinaceous or lipidic nature (Yang et al., 2017), which was consistently downregulated in producers compared with the null pool in the DAVI dataset at both time points (Table 3). In addition, *Cd36* was strongly downregulated in 3068 and 3080, the cell lines with the highest Qp in the JCE dataset, compared with 3936 and 4384 at both Day 4 and Day 7. Heat shock protein family A member 8 (*Hspa8*), a molecular chaperone implicated in the protein quality control system and protection of the proteome from stress (Stricher et al., 2013), was downregulated at Day 12 against Day 4 in the DAVI dataset among every producer cell line and when compared with the null 3478 cell line at Day 12 (Table 3). An identical pattern of downregulation was shown by both *Serpinh1*, a collagen-specific molecular chaperone localized to the ER (Ito and Nagata, 2017), and vimentin (*Vim*), a type III intermediate filament protein responsible for maintaining cell shape and stabilizing cytoskeletal interactions (Musaelyan et al., 2018). Overall, *Cd36, Hspa8, Serpinh1,* and *Vim* were consistently downregulated in cell lines with higher Qp in our datasets in agreement with previous transcriptomics studies summarized in the meta-analysis (Tamošaitis and Smales, 2018).

**Table 3:** Top Four Differentially Expressed Genes Based on Occurrence in the DAVI and JCE Datasets Based on a Recent Meta-Analysis Comparison of Transcriptomics Studies in CHO Cells Linked to Qp and Growth

Dataset	Gene	Time Point	Cell Line	FC	padj
DAVI	Cd36	D12 vs D4	3080	−4.31	8.03 × 10^−15^
		D4	3068 vs 3478	−2.55	2.29 × 10^−3^
		D4	3077 vs 3478	−3.40	2.77 × 10^−8^
		D4	3080 vs 3068	−2.53	5.53 × 10^−3^
		D4	3080 vs 3478	−6.45	2.16 × 10^−25^
		D12	3068 vs 3478	−4.14	1.49 × 10^−13^
		D12	3077 vs 3478	−4.31	8.03 × 10^−15^
		D12	3080 vs 3478	−6.63	1.35 × 10^−27^
JCE	Cd36	D4	3936 vs 3080	5.75	4.62 × 10^−7^
		D4	4384 vs 3068	4.28	6.20 × 10^−6^
		D4	4384 vs 3080	11.33	6.85 × 10^−18^
		D7	3936 vs 3080	6.16	5.89 × 10^−10^
		D7	4384 vs 3068	5.10	3.64 × 10^−8^
		D7	4384 vs 3080	13.44	6.76 × 10^−26^
DAVI	Serpinh1	D12 vs D4	3068	−1.83	6.03 × 10^−3^
		D12 vs D4	3077	−1.85	3.09 × 10^−3^
		D12 vs D4	3080	−2.00	9.49 × 10^−6^
		D12	3068 vs 3478	−1.90	6.06 × 10^−4^
		D12	3077 vs 3478	−2.00	9.49 × 10^−6^
		D12	3080 vs 3478	−2.08	5.98 × 10^−7^
JCE	Serpinh1	D7	3936 vs 3080	2.03	5.96 × 10^−2^
DAVI	Vim	D12 vs D4	3068	−2.47	4.67 × 10^−7^
		D12 vs D4	3077	−3.40	1.30 × 10^−18^
		D12 vs D4	3080	−3.28	6.80 × 10^−17^
		D12	3068 vs 3478	−2.64	9.90 × 10^−9^
		D12	3077 vs 3478	−3.28	6.80 × 10^−17^
		D12	3080 vs 3478	−2.61	3.25 × 10^−8^
JCE	Vim	D4	4384 vs 3080	2.14	1.19 × 10^−2^
		D7	3936 vs 3080	2.73	9.30 × 10^−7^
		D7	4384 vs 3080	2.19	7.49 × 10^−3^
DAVI	Hspa8	D12 vs D4	3068	−3.93	9.49 × 10^−45^
		D12 vs D4	3077	−3.41	2.54 × 10^−32^
		D12 vs D4	3080	−3.90	1.23 × 10^−43^
		D12	3068 vs 3478	−3.59	5.35 × 10^−36^
		D12	3077 vs 3478	−3.90	1.23 × 10^−43^
		D12	3080 vs 3478	−2.18	1.41 × 10^−6^

From left to right, the table indicates the dataset, the gene, the time point, and cell line used in the comparison, the fold-change, and the adjusted p value (Tamošaitis and Smales, 2018).

### Conclusion

In this work we present and make available to the community two RNA-Seq-derived transcriptomic datasets that comprehensively detail coding and non-coding transcript expression analysis of five IgG1-producing CHO cell lines and one null pool at different time points cultivated under fed-batch conditions in an ambr®15 system. In particular, we use RNA-Seq to confirm the expression of lncRNAs in CHO cells and identify those whose expression is differentially regulated throughout fed-batch culture and between cell lines with different characteristics. The different time points for sample collection throughout culture and Qp of cell lines were clearly reflected in the PCA clustering of the transcript expression analysis and in the numbers of differentially expressed genes for the DAVI dataset. In addition, feeding was shown to be a significant source of variability even at early stage of culture, as shown by the comparison of seed train flask data used to inoculate the fed-batch process. These data show that there is a significant change in gene expression after only four days of culture among the same clone when the cells are still rapidly growing and dividing and before the major phase of production of the protein of interest. Taking into account this early transcriptional variability induced by changes in culture conditions, leading to differential regulation of the genome maintenance pathways, could be beneficial for a more effective cell engineering and clone selection in industrial settings.

KEGG functional enrichment analysis confirmed a tendency of pathways in the replication and repair domain to be differentially regulated in response to feeding when the seed train is inoculated in the fed-batch process and toward Day 12 in particular. The only exception was the clone with the highest Qp at Day 12, 3080, which interestingly did not show any differentially regulated pathways in the replication and repair domain. These data suggest that those cell lines that can maintain genome integrity and its surveillance may be better suited to prolonged culture and recombinant protein productivity. Although our datasets contained six different cell lines, we wanted to identify coding genes correlated to an increase in Qp across literature to improve robustness across different systems, leading to the identification of *Cd36*, *Hspa8*, *Serpinh1,* and *Vim* as genes negatively correlated with Qp in both our datasets and the most recent transcriptomics meta-analysis in CHO cells (Tamošaitis and Smales, 2018). Although the functions of those genes are heterogeneous, the conserved pattern of expression among very different experimental settings and cell lines suggests conserved roles with detrimental effects on Qp. Knock-out (KO) or knock-down (KD) strategies on the aforementioned genes with CRISPR or RNA interference could be implemented to investigate and validate any effects (Lee et al., 2015, Wu, 2009).

In conclusion, although many studies in CHO cells have investigated coding genes, our work aimed to unveil the non-coding transcriptome variation, specifically lncRNAs. We identified *Adapt15*, *GAS5*, and *PVT1* among many others as lncRNAs linked to Qp. Although these genes lack an annotation in Chinese hamster genomes, their effects in model organisms and human diseases are well established (Colombo et al., 2015, Hollander et al., 1996, Ma et al., 2016). Although *Adapt15* was initially identified in hamster and later linked to ER stress, DNA damage, and cell death, its role in CHO cells has never been further investigated. *Adapt15* upregulation in producing cell lines toward Day 12 of culture suggests an increasing stress on the ER as the recombinant protein is produced. In addition, we found higher levels of Adapt15 to be correlated to complete dysregulation of the genome maintenance pathways toward the end of culture in the producing clones. Our results suggest *Adapt15* as a target for knock-down with RNAi or knock-out with CRISPR to alleviate its detrimental effects on productivity and genome integrity. *GAS5* transcript accumulation occurs as a result of mTOR signaling repression, suggesting its use as a marker of translation repression and cell growth arrest in specific cell lines. Our data also suggest a correlation between the expression of the lncRNA *PVT1* and of *Myc*; the relationship between the two depends on the stage of fed-batch culture and is reflected in the differential regulation of the PI3K/AKT pathway. When compared with an existent public database of gene expression (Singh et al., 2018), these lncRNAs are constitutively expressed in different CHO-S, CHOK1, and DG44 cell lines, suggesting a conserved functional role that could be exploited for cell engineering. Although lncRNAs investigation presents specific challenges in CHO due to the almost complete lack of annotation in the genome, our workflow allowed the identification, among many, of three strong candidates for cell engineering based on literature, structural predictions, transcriptional expression, and association with coding genes and pathways. Although we were able to propose functional mechanisms of action using secondary structure and RNA-protein interaction predictions, focused experimental studies on single transcripts will be required to assess their effects in mammalian cell factories.

### Limitations of the Study

Although we have confirmed the expression of lncRNAs in CHO cells in this study by RNA-seq and qPCR and shown that the expression of some of these correlates with either cell growth or recombinant protein productivity, the conclusions around the potential impact of changes in the expression of specific lncRNAs that relate to productivity or growth across the different CGO cell lines are, at this stage, based upon correlation analysis only. Such correlation analysis does not imply cause or effect but merrily identifies these lncRNAs as potentially of interest. In order to determine any definitive link between the expression of specific lncRNAs and cell growth or productivity, it would be necessary to show experimentally that manipulation of the identified lncRNAs with expression profiles correlating with these phenotypes impacted the expected phenotype (growth or productivity) by, for example, knockdown/out or over-expression studies.

## Methods

All methods can be found in the accompanying Transparent Methods supplemental file.

## Data and Code Availability

The accession number for the data reported in this paper is Gene Expression Omnibus (GEO) accession number GEO: GSE140671. The direct link to the data is www.ncbi.nlm.nih.gov/projects/geo/query/acc.cgi?acc=GSE140671.

The complete RNA-Seq dataset is thus provided as a reference for the community to investigate RNAseq data from CHO cells (see GEO accession number GEO: GSE140671 and Data S1).

## Supplemental Information

Document S1. Transparent Methods, Figures S1–S6, and Table S1

Data S1. The Complete RNA-Seq Dataset Provided as a Reference for the Community to Investigate RNAseq Data from CHO Cells (See Also GEO Accession Number GSE140671), Related to Figure 2

Data S2. qRT-PCR Analysis of a Representative Group of Differentially Expressed Coding and Non-coding RNAs to Validate RNAseq Data, Related to Figure 3

Data S3. Comparison of the Regulated Expression of Coding Genes in This Study with Those Reported in a Previous Meta-analysis Comparing Transcriptomics Studies in CHO Cells (Tamošaitis & Smales, 2018), Related to Table 3

## References

[bib1] Alsayyari A.A., Pan X., Dalm C., van der Veen J.W., Vriezen N., Hageman J.A., Wijffels R.H., Martens D.E. (2018). Transcriptome analysis for the scale-down of a CHO cell fed-batch process. J. Biotechnol..

[bib2] Bareither R., Pollard D. (2011). A review of advanced small-scale parallel bioreactor technology for accelerated process development: Current state and future need. Biotechnol. Prog..

[bib3] Brookheart R.T., Michel C.I., Listenberger L.L., Ory D.S., Schaffer J.E. (2009). The non-coding RNA gadd7 is a regulator of lipid-induced oxidative and endoplasmic reticulum stress. J. Biol. Chem..

[bib4] Chen C., Le H., Goudar C.T. (2017). Evaluation of two public genome references for Chinese hamster ovary cells in the context of rna-seq based gene expression analysis. Biotechnol. Bioeng..

[bib5] Colombo T., Farina L., Macino G., Paci P. (2015). PVT1: a rising star among oncogenic long noncoding RNAs. Biomed. Res. Int..

[bib6] Crawford D.R., Schools G.P., Salmon S.L., Davies K.J.A. (1996). Hydrogen peroxide induces the expression of adapt15, a novel RNA associated with polysomes in hamster HA-1 cells. Arch. Biochem. Biophys..

[bib7] Djebali S., Davis C.A., Merkel A., Dobin A., Lassmann T., Mortazavi A., Tanzer A., Lagarde J., Lin W., Schlesinger F. (2012). Landscape of transcription in human cells. Nature.

[bib8] Durocher Y., Butler M. (2009). Expression systems for therapeutic glycoprotein production. Curr. Opin. Biotechnol..

[bib9] Faustrup Kildegaard H., Baycin-Hizal D., Lewis N.E., Betenbaugh M.J. (2013). The emerging CHO systems biology era: harnessing the ’omics revolution for biotechnology. Curr. Opin. Biotechnol..

[bib10] Geisler S., Coller J. (2013). RNA in unexpected places: long non-coding RNA functions in diverse cellular contexts. Nat. Rev. Mol. Cell Biol..

[bib11] Godfrey C.L., Mead E.J., Daramola O., Dunn S., Hatton D., Field R., Pettman G., Smales C.M. (2017). Polysome profiling of mAb producing CHO cell lines links translational control of cell proliferation and recombinant mRNA loading onto ribosomes with global and recombinant protein synthesis. Biotechnol. J..

[bib12] Hollander M.C., Alamo I., Fornace A.J. (1996). A novel DNA damage-inducible transcript, gadd7, inhibits cell growth, but lacks a protein product. Nucleic Acids Res..

[bib13] Ito S., Nagata K. (2017). Biology of Hsp47 (Serpin H1), a collagen-specific molecular chaperone. Semin. Cell Dev. Biol..

[bib14] Janakiraman V., Kwiatkowski C., Kshirsagar R., Ryll T., Huang Y.-M. (2015). Application of high-throughput mini-bioreactor system for systematic scale-down modeling, process characterization, and control strategy development. Biotechnol. Prog..

[bib15] Jossé L., Zhang L., Smales C.M. (2018). Application of microRNA targeted 3′UTRs to repress DHFR selection marker expression for development of recombinant antibody expressing CHO cell pools. Biotechnol. J..

[bib16] Kalvari I., Argasinska J., Quinones-Olvera N., Nawrocki E.P., Rivas E., Eddy S.R., Bateman A., Finn R.D., Petrov A.I. (2018). Rfam 13.0: shifting to a genome-centric resource for non-coding RNA families. Nucleic Acids Res..

[bib17] Kang Y.-J., Yang D.-C., Kong L., Hou M., Meng Y.-Q., Wei L., Gao G. (2017). CPC2: a fast and accurate coding potential calculator based on sequence intrinsic features. Nucleic Acids Res..

[bib18] Kashi K., Henderson L., Bonetti A., Carninci P. (2016). Discovery and functional analysis of lncRNAs: methodologies to investigate an uncharacterized transcriptome. Biochim. Biophys. Acta.

[bib19] Kopp F., Mendell J.T. (2018). Functional classification and experimental dissection of Long noncoding RNAs. Cell.

[bib20] Kunert R., Reinhart D. (2016). Advances in recombinant antibody manufacturing. Appl. Microbiol. Biotechnol..

[bib21] Kung J.T.Y., Colognori D., Lee J.T. (2013). Long noncoding RNAs: past, present, and future. Genetics.

[bib22] Lee J.S., Grav L.M., Lewis N.E., Faustrup Kildegaard H. (2015). CRISPR/Cas9-mediated genome engineering of CHO cell factories: Application and perspectives. Biotechnol. J..

[bib23] Leu J.I.-J., Dumont P., Hafey M., Murphy M.E., George D.L. (2004). Mitochondrial p53 activates Bak and causes disruption of a Bak–Mcl1 complex. Nat. Cell Biol..

[bib24] Lewis N.E., Liu X., Li Y., Nagarajan H., Yerganian G., O’Brien E., Bordbar A., Roth A.M., Rosenbloom J., Bian C. (2013). Genomic landscapes of Chinese hamster ovary cell lines as revealed by the *Cricetulus griseus* draft genome. Nat. Biotechnol..

[bib25] Love M.I., Huber W., Anders S. (2014). Moderated estimation of fold change and dispersion for RNA-seq data with DESeq2. Genome Biol..

[bib26] Lu Q., Ren S., Lu M., Zhang Y., Zhu D., Zhang X., Li T. (2013). Computational prediction of associations between long non-coding RNAs and proteins. BMC Genomics.

[bib27] Ma C., Shi X., Zhu Q., Li Q., Liu Y., Yao Y., Song Y. (2016). The growth arrest-specific transcript 5 (GAS5): a pivotal tumor suppressor long noncoding RNA in human cancers. Tumor Biol..

[bib28] Mead E.J., Chiverton L.M., Smales C.M., Von Der Haar T. (2009). Identification of the limitations on recombinant gene expression in CHO cell lines with varying luciferase production rates. Biotechnol. Bioeng..

[bib29] Mead E.J., Masterton R.J., Feary M., Obrezanova O., Zhang L., Young R., Smales C.M. (2015). Biological insights into the expression of translation initiation factors from recombinant CHOK1SV cell lines and their relationship to enhanced productivity. Biochem. J..

[bib30] Muppirala U.K., Honavar V.G., Dobbs D. (2011). Predicting RNA-protein interactions using only sequence information. BMC Bioinformatics.

[bib31] Musaelyan A., Lapin S., Nazarov V., Tkachenko O., Gilburd B., Mazing A., Mikhailova L., Shoenfeld Y. (2018). Vimentin as antigenic target in autoimmunity: a comprehensive review. Autoimmun. Rev..

[bib32] Nienow A.W., Rielly C.D., Brosnan K., Bargh N., Lee K., Coopman K., Hewitt C.J. (2013). The physical characterisation of a microscale parallel bioreactor platform with an industrial CHO cell line expressing an IgG4. Biochem. Eng. J..

[bib33] Pan X., Streefland M., Dalm C., Wijffels R.H., Martens D.E. (2017). Selection of chemically defined media for CHO cell fed-batch culture processes. Cytotechnology.

[bib34] Patrucco L., Chiesa A., Soluri M.F., Fasolo F., Takahashi H., Carninci P., Zucchelli S., Santoro C., Gustincich S., Sblattero D., Cotella D. (2015). Engineering mammalian cell factories with SINEUP noncoding RNAs to improve translation of secreted proteins. Gene.

[bib35] Perry R.B.-T., Ulitsky I. (2016). The functions of long noncoding RNAs in development and stem cells. Development.

[bib36] Povey J.F., O’Malley C.J., Root T., Martin E.B., Montague G.A., Feary M., Trim C., Lang D.A., Alldread R., Racher A.J., Smales C.M. (2014). Rapid high-throughput characterisation, classification and selection of recombinant mammalian cell line phenotypes using intact cell MALDI-ToF mass spectrometry fingerprinting and PLS-DA modelling. J. Biotechnol..

[bib37] Rouiller Y., Bielser J.-M., Brühlmann D., Jordan M., Broly H., Stettler M. (2016). Screening and assessment of performance and molecule quality attributes of industrial cell lines across different fed-batch systems. Biotechnol. Prog..

[bib60] Sandner V., Pybus L.P., McCreath G., Glassey J. (2019). Scale-down model development in ambr systems: an industrial perspective. Biotechnol. J..

[bib38] Schmitt A.M., Chang H.Y. (2016). Long noncoding RNAs in cancer pathways. Cancer Cell.

[bib39] Singh A., Kildegaard H.F., Andersen M.R. (2018). An online compendium of CHO RNA-Seq data allows identification of CHO cell line-specific transcriptomic signatures. Biotechnol. J..

[bib40] Smith C.M., Steitz J.A. (1998). Classification of gas5 as a multi-small-nucleolar-RNA (snoRNA) host gene and a member of the 5’-terminal oligopyrimidine gene family reveals common features of snoRNA host genes. Mol. Cell Biol..

[bib41] Stricher F., Macri C., Ruff M., Muller S. (2013). HSPA8/HSC70 chaperone protein. Autophagy.

[bib42] Sun Z.-Y., Jian Y.-K., Zhu H.-Y., Li B. (2019). lncRNAPVT1 targets miR-152 to enhance chemoresistance of osteosarcoma to gemcitabine through activating c-MET/PI3K/AKT pathway. Pathol. Res. Pract..

[bib43] Tamošaitis L., Smales C.M. (2018). Meta-analysis of publicly available Chinese Hamster Ovary (CHO) cell transcriptomic datasets for identifying engineering targets to enhance recombinant protein yields. Biotechnol. J..

[bib44] Tani H., Torimura M., Akimitsu N. (2013). The RNA degradation pathway regulates the function of GAS5 a non-coding RNA in mammalian cells. PLoS One.

[bib45] Tseng Y.-Y., Bagchi A. (2015). The PVT1-MYC duet in cancer. Mol. Cell. Oncol..

[bib46] Tseng Y.-Y., Moriarity B.S., Gong W., Akiyama R., Tiwari A., Kawakami H., Ronning P., Reuland B., Guenther K., Beadnell T.C. (2014). PVT1 dependence in cancer with MYC copy-number increase. Nature.

[bib47] Urlaub G., Käs E., Carothers A.M., Chasin L.A. (1983). Deletion of the diploid dihydrofolate reductase locus from cultured mammalian cells. Cell.

[bib48] Vishwanathan N., Le H., Le T., Hu W.S. (2014). Advancing biopharmaceutical process science through transcriptome analysis. Curr. Opin. Biotechnol..

[bib49] Vito D., Smales C.M. (2018). The Long non-coding RNA transcriptome landscape in CHO cells under batch and fed-batch conditions. Biotechnol. J..

[bib50] Walsh G. (2010). Biopharmaceutical benchmarks 2010. Nat. Biotechnol..

[bib51] Wilusz J.E. (2016). Long noncoding RNAs: re-writing dogmas of RNA processing and stability. Biochim. Biophys. Acta.

[bib52] Wong D.C.F., Wong K.T.K., Lee Y.Y., Morin P.N., Heng C.K., Yap M.G.S. (2006). Transcriptional profiling of apoptotic pathways in batch and fed-batch CHO cell cultures. Biotechnol. Bioeng..

[bib53] Wu S.-C. (2009). RNA interference technology to improve recombinant protein production in Chinese hamster ovary cells. Biotechnol. Adv..

[bib54] Wurm F.M. (2013). CHO quasispecies—implications for manufacturing processes. Processes.

[bib55] Wurm F., Wurm M. (2017). Cloning of CHO cells, productivity and genetic stability—a discussion. Processes.

[bib56] Xu X., Nagarajan H., Lewis N.E., Pan S., Cai Z., Liu X., Chen W., Xie M., Wang W., Hammond S. (2011). The genomic sequence of the Chinese hamster ovary (CHO)-K1 cell line. Nat. Biotechnol..

[bib57] Yang X., Okamura D.M., Lu X., Chen Y., Moorhead J., Varghese Z., Ruan X.Z. (2017). CD36 in chronic kidney disease: novel insights and therapeutic opportunities. Nat. Rev. Nephrol..

[bib58] Zhu S., Shuai P., Yang C., Zhang Y., Zhong S., Liu X., Chen K., Ran Q., Yang H., Zhou Y. (2017). Prognostic value of long non-coding RNA PVT1 as a novel biomarker in various cancers: a meta-analysis. Oncotarget.

[bib59] Zucchelli S., Patrucco L., Persichetti F., Gustincich S., Cotella D. (2016). Engineering translation in mammalian cell factories to increase protein yield: the unexpected use of Long non-coding SINEUP RNAs. Comput. Struct. Biotechnol. J..

