# Supplementary material for: Defining lncRNAs Correlated with CHO Cell Growth and IgG Productivity by RNA-Seq
Source: iScience. 2019 Dec 18;23(1):100785. doi: 10.1016/j.isci.2019.100785 (PMC6971398; doi:10.1016/j.isci.2019.100785)
Supplement: Document S1. Transparent Methods, Figures S1–S6, and Table S1 [file mmc1.pdf]

iScience, Volume *23*

Supplemental Information

Defining lncRNAs Correlated with CHO Cell Growth and IgG Productivity by RNA-Seq

Davide Vito, Jens Christian Eriksen, Christian Skjødt, Dietmar Weilguny, Søren K. Rasmussen, and C. Mark Smales

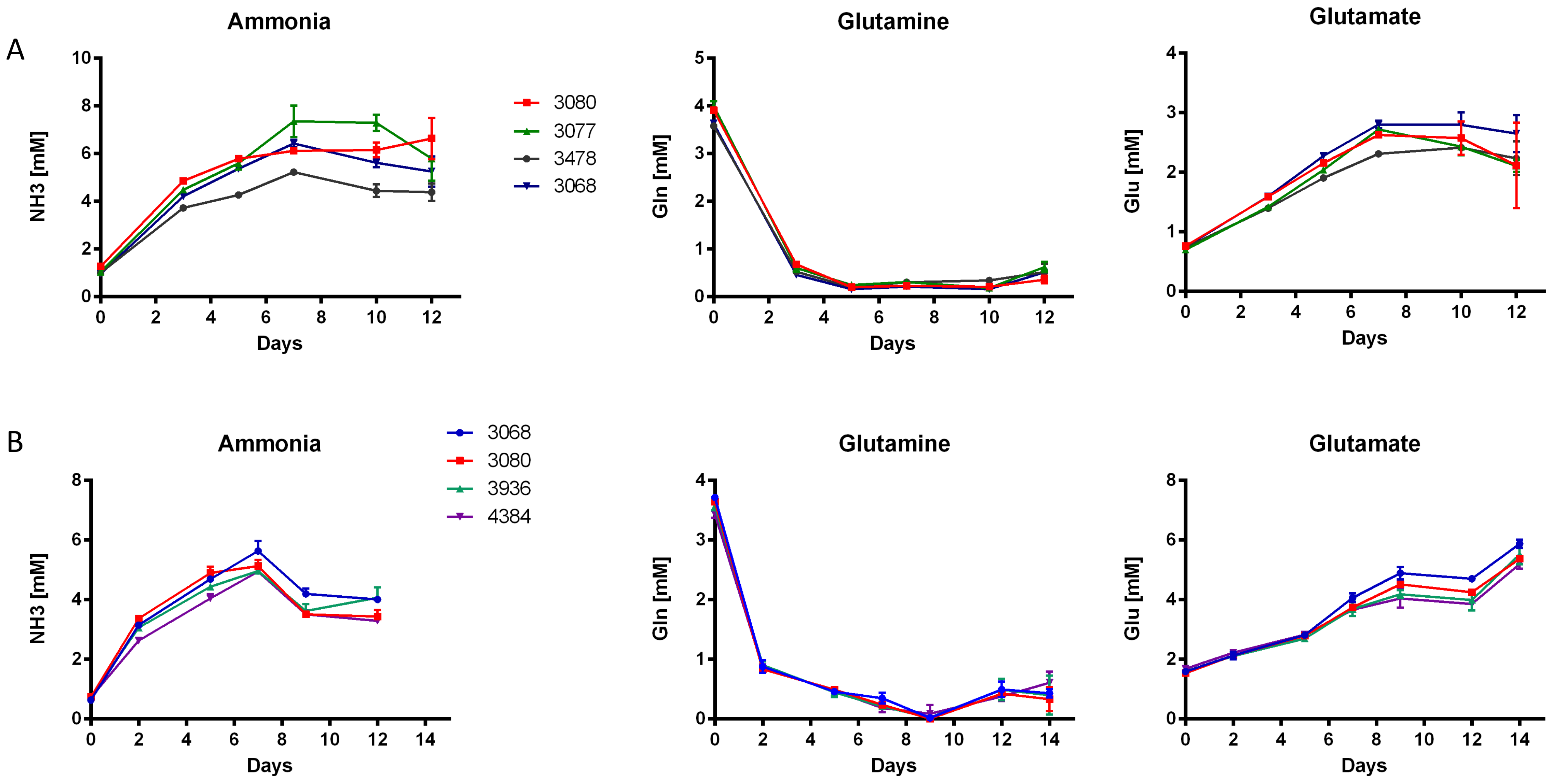

Figure S1. Ammonia, glutamine and glutamate concentrations over time for the DAVI dataset (panel A) and the JCE dataset (panel B). Data are represented as mean +/- SEM, Related to Figure 1.

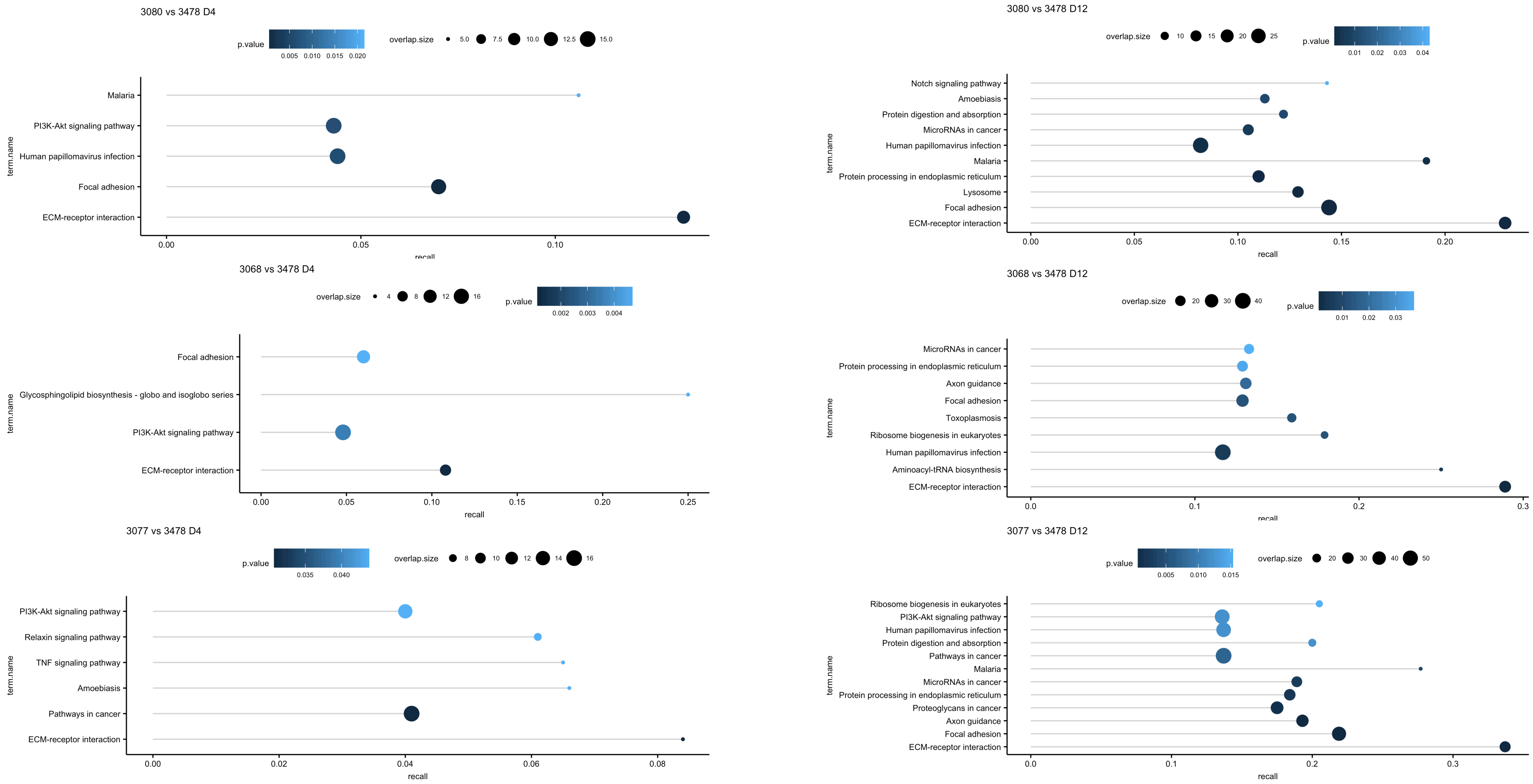

Figure S2. Enriched KEGG pathways based on differentially expressed genes for each comparison between different cell lines at the same time point in the DAVI dataset. Each dot represents a pathway, with color shade representing the *p*-value, size proportional to the overlap size (differentially expressed genes in the pathway) and x-coordinate recall (overlap size divided by the total number of genes in the pathway), Related to Figure 4.

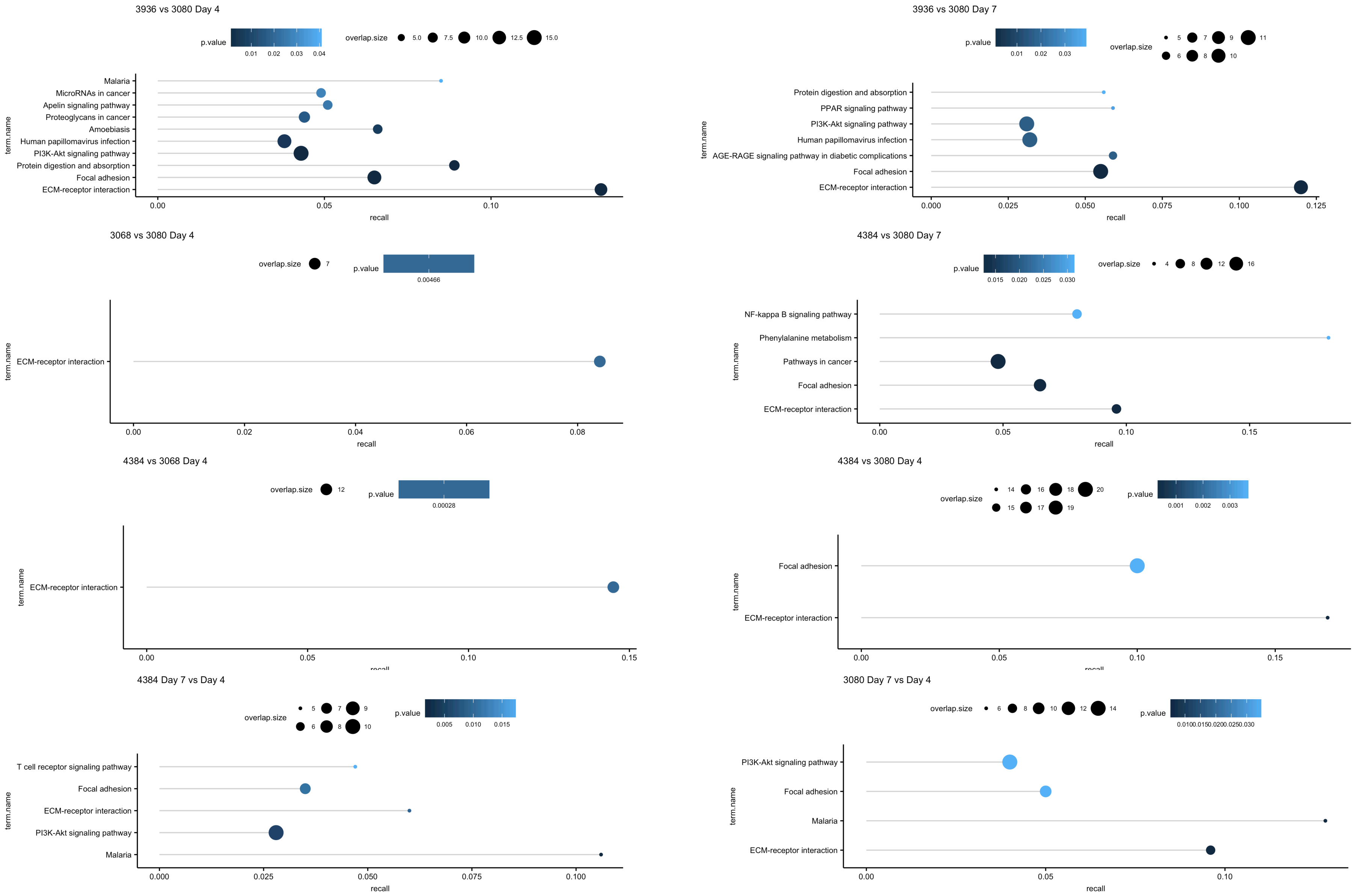

Figure S3. Enriched KEGG pathways based on differentially expressed genes for each comparison among different cell lines at day 4 and day 7 in the JCE dataset. Each dot represents a pathway, with color shade representing the *p*-value, size proportional to the overlap size (differentially expressed genes in the pathway) and x-coordinate recall (overlap size divided by the total number of genes in the pathway), Related to Figure 4.

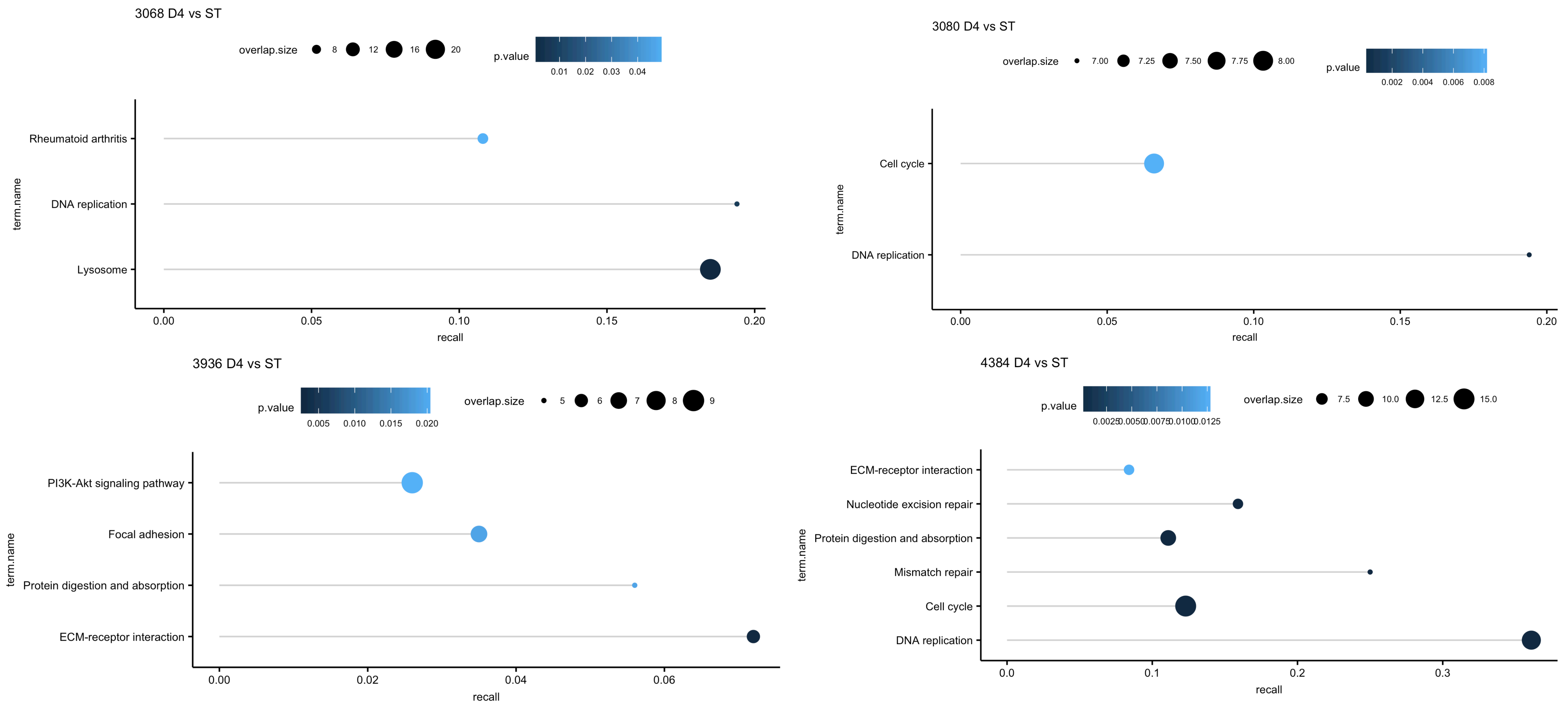

Figure S4. Enriched KEGG pathways based on differentially expressed genes for each comparison among the same cell line at day 4 against seed train culture in the JCE dataset. Each dot represents a pathway, with color shade representing the *p*-value, size proportional to the overlap size (differentially expressed genes in the pathway) and x-coordinate recall (overlap size divided by the total number of genes in the pathway), Related to Figure 4.

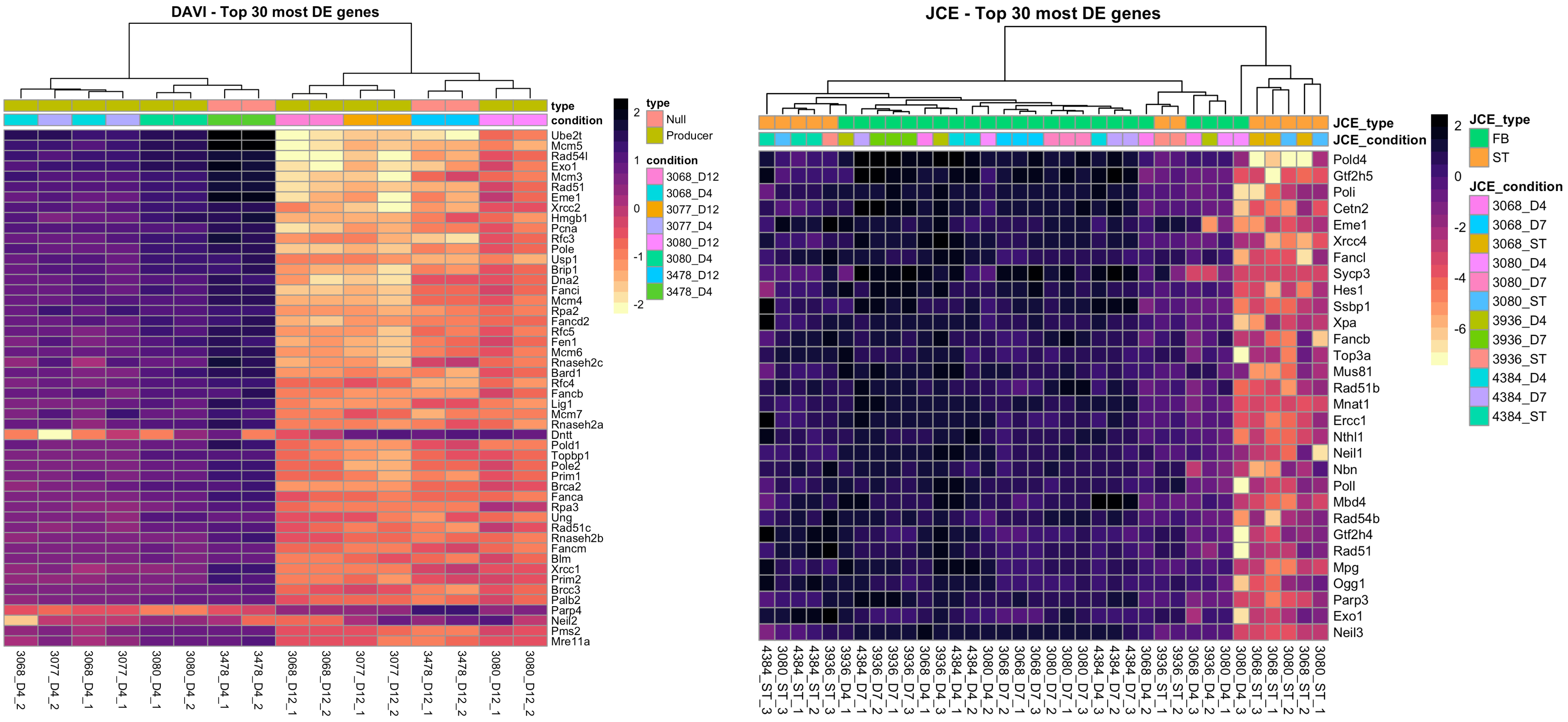

Figure S5. Top 30 differentially expressed genes among the replication and repair domain in the DAVI dataset (A) and JCE dataset (B), Related to Figure 4.

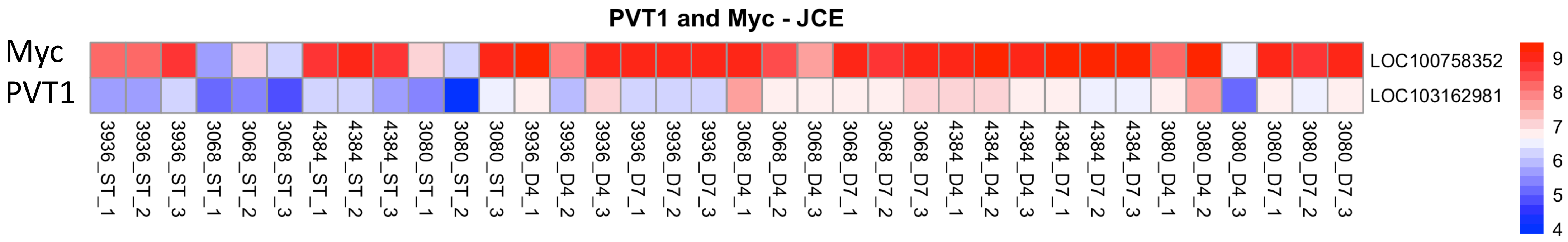

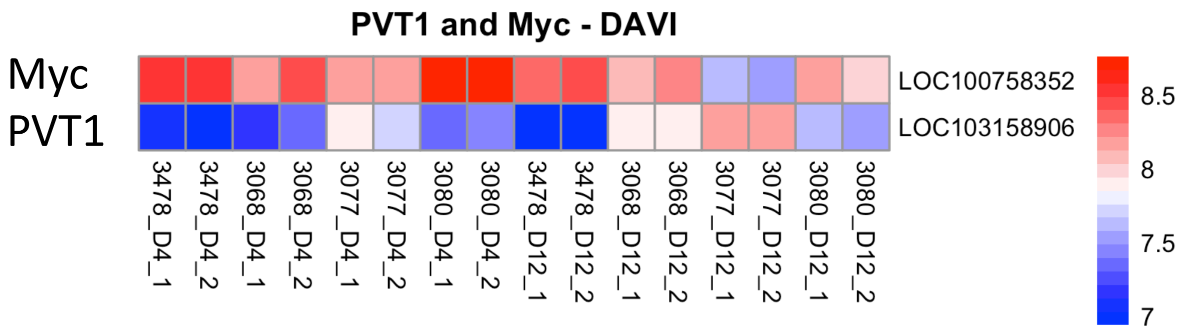

Figure S6. PVT1 and Myc expression in the JCE (top) and DAVI (bottom) datasets, Related to Figure 5.

Table S1. The table shows the number of differentially expressed genes with adj *p*-value < 0.10 for each comparison among the DAVI and JCE datasets, with the percentage of ncRNAs genes, Related to Figure 3.

Comparison	DE Genes	Dataset	ncRNAs [%]
**3068 v 3478 Day 4**	371	DAVI	32.1
**3077 v 3478 Day 4**	509	DAVI	27.9
**3080 v 3478 Day 4**	491	DAVI	26.3
**3080 v 3068 Day 4**	570	DAVI	30.2
**3077 v 3068 Day 4**	223	DAVI	25.6
**3077 v 3080 Day 4**	604	DAVI	30.0
**3068 v 3478 Day 12**	1653	DAVI	14.6
**3077 v 3478 Day 12**	2255	DAVI	15.4
**3080 v 3478 Day 12**	997	DAVI	19.0
**3080 v 3068 Day 12**	813	DAVI	25.7
**3077 v 3068 Day 12**	280	DAVI	22.1
**3077 v 3080 Day 12**	883	DAVI	26.5
**3478 Day 12 v Day 4**	4464	DAVI	13.0
**3068 Day 12 v Day 4**	2294	DAVI	16.3
**3077 Day 12 v Day 4**	2538	DAVI	14.5
**3080 Day 12 v Day 4**	2255	DAVI	15.4
**3068 Day 7 v Day 4**	417	JCE	8.4
**3080 Day 7 v Day 4**	516	JCE	9.1
**3936 Day 7 v Day 4**	615	JCE	8.6
**4384 Day 7 v Day 4**	181	JCE	8.3
**3068 v 3080 Day 4**	301	JCE	20.3
**3068 v 3936 Day 4**	170	JCE	19.4
**3936 v 3080 Day 4**	316	JCE	17.7
**4384 v 3068 Day 4**	701	JCE	10.6
**4384 v 3080 Day 4**	1015	JCE	12.6
**4384 v 3936 Day 4**	472	JCE	13.3
**3068 v 3080 Day 7**	220	JCE	20.9
**3068 v 3936 Day 7**	152	JCE	21.1
**3936 v 3080 Day 7**	228	JCE	14.9
**4384 v 3068 Day 7**	489	JCE	12.1
**4384 v 3080 Day 7**	525	JCE	13.5
**4384 v 3936 Day 7**	314	JCE	16.9
**3936 FB Day 4 v ST**	140	JCE	15.7
**4384 FB Day 4 v ST**	306	JCE	9.8
**3080 FB Day 4 v ST**	307	JCE	15.6
**3068 FB Day 4 v ST**	761	JCE	10.1

Transparent Methods

Cell culture Four Symphogen in-house mAb CHO cell clones (designated 3068, 3077, 3080, 4384 producing the same IgG subtype) and two cell pools, of which one (3936) was IgG1-producing and the other (3478) was a null-producer, were used in this study. All were generated from a modified dihydrofolate reductase-deficient (DHFR-) CHO DG44 host cell line (Urlaub, Käs, Carothers, & Chasin, 1983) through transfection with a vector containing the DHFR gene and the genes for the antibody heavy (HC) and light chains (LC) and methotrexate (MTX) mediated stable selection . Clones were isolated by fluorescence activated cell sorting (FACS). Cells were routinely maintained and expanded in PowerCHO-2 CD (Lonza) as basal media in shake flasks with shaking at 190-200 rpm in a 37°C humidified culture incubator supplied with 5% CO_2.

For upstream experiments to generate samples for analysis, cells were inoculated at a starting concentration of either 0.4x 10^6 viable cells/ml (DAVI experiments) or 0.6 x 10^6 viable cells/ml (JCE experiments), in a total culture volume of 13 ml. The ambr15™ micro bioreactor by Sartorius (Goettingen, Germany) was used to run fed-batch culture experiments for either 12 (DAVI) or 14 days (JCE). For feeding of cultures, HyClone Cell Boost 6 Supplement (GE Healthcare Bio-Sciences AB, Uppsala, Sweden) + 8% FunctionMAX™ TiterEnhancer (ThermoFisher Scientific, Massachusetts, United States) was used with a feeding regime of every 2nd or 3rd day for JCE experiments or daily from the 2nd day of culture for DAVI experiments. Culture viability and viable cell concentration (VCD) was measured using a Vi-CELL XR (Beckman Coulter, Brea, CA) instrument. For JCE experiments, glucose, glutamine, lactate, ammonium, glutamate, pH, and osmolality were measured using a Bioprofile 100plus (Nova BioMedical, Waltham, WA) instrument while IgG titer was determined by biolayer interferometry using an Octet QK384 instrument equipped with Protein G biosensors (ForteBio, Menlo Park, CA). For DAVI experiments, a Cedex Bio HT analyser (Roche, Basel, Switzerland) was used for all measurements.

Sampling from Cell Cultures and Subsequent RNA Extraction For seed train samples, cells were harvested 48 hours after adjusting their VCD to 0.3x10^6 viable cells/ml, when a steady doubling time

and high culture viability were maintained. Cell suspensions from each seed-train in the JCE dataset were added to 15 ml Falcon tubes and centrifuged at 1000 rpm for 4 minutes, followed by Direct-zol RNA extraction (Zymo Research, California, USA) following the manufacturer's instructions. For samples taken from the fed-batch cultures, 1 x 10^7 viable cells were taken before feeding from each biological replicate ambr15™ bioreactor after 96 h of culture time (day 4, both DAVI and JCE experiments) and after either 168 h (day 7, JCE experiment) or 288 h (day 12, DAVI experiment) of culture. Cells were lysed using TRI-Reagent (Sigma-Aldrich, Missouri, USA) followed by extraction of total RNA using a Direct-zol RNA Kit (Zymo Research) and in-column DNAse treated. The RNA quantity/quality was determined using a NanoDrop instrument (ThermoFisher Scientific) and RNA 6000 Pico Kit (Agilent, California, USA).

RNA Seq and Data analysis For DAVI experiments: the RNA was sent to the Oxford Genomics Centre (Oxford, UK) where the Ribo-Zero ribosomal RNA (rRNA) removal kit (Illumina, California, USA) was used to remove rRNAs followed by sequencing on an HiSeq4000 by Illumina. The fastq files were initially checked for quality using FastQC, sorted using samtools, deduplicated using Picard MarkDuplicates then aligned with HISAT2 to the CriGri_1.0 reference genome. The gene counts were calculated using featureCounts, then the differential expression analysis was undertaken with the R/Bioconductor package DESeq2 (Love, Huber, & Anders, 2014).

For JCE experiments: samples were sequenced at Genotypic (Bengaluru, India). The fastq files were trimmed using Trim_Galore! and aligned to GCF_000419365.1 with STAR. The gene counts were calculated using featureCounts, then the differential expression analysis was conducted with the R package DESeq2 (Love et al., 2014) as described for the DAVI experiments. Genes with a fold change (FC) > 1.50 and Benjamini-Hochberg adjusted *p*-value <0.10 in both datasets were selected for GO and KEGG enrichment using the Bioconductor package gProfileR (Data S1).

RT-qPCR Validation of Differentially Expressed genes Primers for RT-qPCR experiments were designed using Primer-BLAST and synthetized by Integrated DNA Technologies (Illinois, USA). Primers used during this study are described in Data S2. RT-qPCR reactions were conducted using a

Mastercycler EP Realplex instrument (Eppendorf, Hamburg, Germany) using the PrecisionPLUS Onestep qRT-PCR Master Mix kit by Primer Design (Southampton, UK). The specificity of amplification was checked by the generation of Tm curves and by analysis of the reaction products using 2% agarose gel electrophoresis to confirm the presence of a single amplicon of the expected size. The results were analysed applying the standard ΔCt method and normalized to β-actin and B2M housekeeping genes expression.
